# Zinc sulfate in combination with a zinc ionophore may improve outcomes in hospitalized COVID-19 patients

**DOI:** 10.1099/jmm.0.001250

**Published:** 2020-09-15

**Authors:** Philip M. Carlucci, Tania Ahuja, Christopher Petrilli, Harish Rajagopalan, Simon Jones, Joseph Rahimian

**Affiliations:** ^1^​ New York University Grossman School of Medicine, Department of Medicine, New York, NY, USA; ^2^​ New York University Langone Health, Department of Pharmacy, New York, NY, USA; ^3^​ NYU Langone Health, New York, NY, USA; ^4^​ Division of Healthcare Delivery Science, Department of Population Health, NYU Grossman School of Medicine, New York, NY, USA; ^5^​ Center for Healthcare Innovation and Delivery Science, NYU Langone Health, New York, NY, USA

**Keywords:** covid 19, coronavirus, hydroxychloroquine, ionophore, mortality, zinc

## Abstract

**Introduction:**

COVID-19 has rapidly emerged as a pandemic infection that has caused significant mortality and economic losses. Potential therapies and prophylaxis against COVID-19 are urgently needed to combat this novel infection. As a result of *in vitro* evidence suggesting zinc sulphate may be efficacious against COVID-19, our hospitals began using zinc sulphate as add-on therapy to hydroxychloroquine and azithromycin.

**Aim:**

To compare outcomes among hospitalized COVID-19 patients ordered to receive hydroxychloroquine and azithromycin plus zinc sulphate versus hydroxychloroquine and azithromycin alone.

**Methodology:**

This was a retrospective observational study. Data was collected from medical records for all patients with admission dates ranging from 2 March 2020 through to 11 April 2020. Initial clinical characteristics on presentation, medications given during the hospitalization, and hospital outcomes were recorded. The study included patients admitted to any of four acute care NYU Langone Health Hospitals in New York City. Patients included were admitted to the hospital with at least one positive COVID-19 test and had completed their hospitalization. Patients were excluded from the study if they were never admitted to the hospital or if there was an order for other investigational therapies for COVID-19.

**Results:**

Patients taking zinc sulphate in addition to hydroxychloroquine and azithromycin (*n*=411) and patients taking hydroxychloroquine and azithromycin alone (*n*=521) did not differ in age, race, sex, tobacco use or relevant comorbidities. The addition of zinc sulphate did not impact the length of hospitalization, duration of ventilation or intensive care unit (ICU) duration. In univariate analyses, zinc sulphate increased the frequency of patients being discharged home, and decreased the need for ventilation, admission to the ICU and mortality or transfer to hospice for patients who were never admitted to the ICU. After adjusting for the time at which zinc sulphate was added to our protocol, an increased frequency of being discharged home (OR 1.53, 95 % CI 1.12–2.09) and reduction in mortality or transfer to hospice among patients who did not require ICU level of care remained significant (OR 0.449, 95 % CI 0.271–0.744).

**Conclusion:**

This study provides the first *in vivo* evidence that zinc sulphate may play a role in therapeutic management for COVID-19.

## Introduction

The World Health Organization has declared a pandemic due to the spread of the coronavirus disease of 2019 (COVID-19) caused by the severe acute respiratory syndrome coronavirus 2 (SARS-CoV2) [1, 2]. Despite limited and conflicting data, the U.S. Food and Drug Administration authorized the emergency use of hydroxychloroquine for the treatment of COVID-19 with or without azithromycin. Hydroxychloroquine was thought to be efficacious partly based on *in vitro* activity against SARS-CoV-2 [3]. However, clinical data in humans has yielded mixed and disappointing results [4–7]. In spite of this, hydroxychloroquine may still have a role at blocking coronavirus replication, when used in combination with zinc, which inhibits coronavirus RNA polymerase activity.

Zinc is an essential trace element that is required for the maintenance of adaptive and innate immune responses [8]. The benefits of zinc have previously been recognized for its therapeutic use against other respiratory viruses including those that cause the common cold [9–11]. Zinc has also been observed to improve pneumonia in children and its deficiency is associated with pneumonia in the elderly [9, 12, 13]. Implicating a role for zinc in COVID-19, zinc inhibits viral RNA-dependent RNA polymerase, and has been shown to do this *in vitro* against SARS-CoV [14]. When combined with a zinc ionophore, such as hydroxychloroquine, cellular uptake is increased making it more likely to achieve suitably elevated intracellular zinc concentrations for viral inhibition [10, 15]. This combination is already being tested as a prophylactic regimen in a prospectively followed cohort (NCT04326725) and in a randomized clinical trial (NCT04377646). Other trials are also investigating this regimen for therapeutic efficacy (NCT04370782, NCT04373733).

As New York became the epicentre of the pandemic, hospitals in the area quickly adopted investigational therapies, including the use of hydroxychloroquine and azithromycin. Given this proposed synergistic effect of zinc with hydroxychloroquine, practices at NYULH changed and the addition of zinc sulphate 220 mg PO BID along with hydroxychloroquine 400 mg once followed by 200 mg PO BID with azithromycin 500 mg once daily became part of the treatment approach for patients admitted to the hospital with COVID-19. This study sought to investigate outcomes among patients who received hydroxychloroquine and azithromycin alone compared to those who received triple therapy of hydroxychloroquine and azithromycin plus zinc sulphate.

## Methods

We performed a retrospective analysis of data from patients hospitalized with confirmed SARS-CoV-2 infection at NYU Langone Health. Data was collected from electronic medical records (Epic Systems, Verona, WI, USA) for all patients being treated with admission dates ranging from 2 March 2020 through 11 April 2020. Patients were admitted to any of four acute care NYU Langone Health hospitals across New York City. COVID-19 positivity was determined by real-time reverse-transcriptase PCR (RT-PCR) of nasopharyngeal or oropharyngeal swabs.

Patients were included in the study if they were admitted to the hospital, had at least one positive test for COVID-19, were ordered to receive hydroxychloroquine and azithromycin, and had either been discharged from the hospital, transitioned to hospice or expired. Patients were excluded from the study if they were never admitted to the hospital or if there was an order for other investigational therapies for COVID-19, including tocilizumab, nitazoxanide, rituximab, anakinra, remdesivir or lopinavir/ritonavir during the course of their hospitalization to avoid potential confounding effects of these medications. We collected demographics as reported by the patient and any past medical history of hypertension, hyperlipidemia, coronary artery disease, heart failure, chronic obstructive pulmonary disease, asthma, malignancy other than non-melanoma skin malignancy and diabetes. We also recorded vital signs on admission, the first set of laboratory results as continuous variables, and relevant medications as categorical variables, including NSAIDs, anticoagulants, antihypertensive medications and corticosteroids ordered at any point during the course of the hospitalization. We compared clinical outcomes of patients who received a combination of zinc sulphate plus hydroxychloroquine, a zinc ionophore, to those that received hydroxychloroquine without zinc supplementation.

### Statistics

Patients were categorized based on their exposure to hydroxychloroquine (400 mg load followed by 200 mg twice daily for 5 days) and azithromycin (500 mg once daily) alone or with zinc sulphate (220 mg capsule containing 50 mg elemental zinc twice daily for 5 days) as treatment in addition to standard supportive care. Descriptive statistics are presented as mean and standard deviation or mean and interquartile range for continuous variables and frequencies for categorical variables. Normality of distribution for continuous variables was assessed by measures of skewness and kurtosis, deeming the dataset appropriate for parametric or nonparametric analysis. A two-tailed Student’s *t*-test was used for parametric analysis, and a Mann–Whitney U test was used for nonparametric data analysis. Pearson’s chi-squared test was used to compare categorical characteristics between the two groups of patients. Linear regression for continuous variables or logistic regression for categorical variables was performed with the presence of zinc as the predictor variable and outcome measures [duration of hospital stay, duration of mechanical ventilation, maximum oxygen flow rate, average oxygen flow rate, average FiO2, maximum FiO2, admission to the intensive care unit (ICU), duration of ICU stay, death/hospice, need for intubation, and discharge destination], as dependent variables. Data was log transformed where appropriate to render the distribution normal for linear regression analysis. Multivariate logistic regression was used to adjust for the timing that our protocol changed to include zinc therapy using admission before or after 25 March as a categorical variable. *P* values less than 0.05 were considered to be significant. All analyses were performed using STATA/SE 16.0 software (STATA Corp.).

### Study approval

The study was approved by the NYU Grossman School of Medicine Institutional Review Board. A waiver of informed consent and a waiver of the Health Information Portability Privacy act were granted.

## Results

Patients taking zinc sulphate in addition to hydroxychloroquine and azithromycin (*n*=411) and patients taking hydroxychloroquine and azithromycin alone (*n*=521) did not differ in age, race, sex, tobacco use or past medical history (Table 1). On hospital admission, vital signs differed by respiratory rate and baseline systolic blood pressure. The first laboratory measurements of inflammatory markers including white blood cell count, absolute neutrophil count, ferritin, d-dimer, creatine phosphokinase, creatinine and C-reactive protein did not differ between groups. Patients treated with zinc sulphate had higher baseline absolute lymphocyte counts [median (IQR), zinc: 1 (0.7–1.3) vs. no zinc: 0.9(0.6–1.3), *P* value: 0.0180] while patients who did not receive zinc had higher baseline troponin [0.01 (0.01–0.02) vs. 0.015 (0.01–0.02), *P* value: 0.0111] and procalcitonin [0.12 (0.05–0.25) vs 0.12 (0.06–0.43), *P* value: 0.0493] (Table 1).

**Table 1.** Comparisons of baseline characteristics and hospital medications. Data are represented as median (IQR) or mean±sd. Sample size is reported where it differed due to lab results not tested. *P* values were calculated using two-sided *t*-test for parametric variables and Mann–Whitney U test for nonparametric continuous variables. Pearson's *χ* ^2^ test was used for categorical comparisons. *P* < 0.05 was deemed significant. Laboratory results represent the first measured value while hospitalized

	Zinc *N*=411	No zinc *N*=521	*P* value
**Demographics**			
Age	63.19±15.18	61.83±15.97	0.0942
Female Sex	147 (35.7 %)	201 (38.6 %)	0.378
Race			0.428
African American	68 (16.5 %)	81 (15.5 %)	
White	189 (46.0 %	244 (46.8 %)	
Asian	30 (7.3 %)	30 (5.8 %)	
Other	97 (23.6 %)	142 (27.2 %)	
Multiracial/Unknown	27 (6.6 %)	24 (4.6 %)	
**History**			
Tobacco use			0.142
Never or Unknown	306 (74.5 %)	382 (73.3 %)	
Former	76 (18.5 %)	115 (22.1 %)	
Current	29 (7.1 %)	24 (4.6 %)	
Any cardiovascular condition	182 (44.3 %)	248 (47.6 %)	0.313
Hypertension	154 (37.5 %)	208 (39.9 %)	0.445
Hyperlipidemia	99 (24.1 %)	148 (28.4 %)	0.138
Coronary Artery Disease	36 (8.8 %)	41 (7.9 %)	0.624
Heart Failure	26 (6.3 %)	22 (4.2 %)	0.149
Asthma or COPD	50 (12.2 %)	56 (10.7 %)	0.499
Diabetes	105 (25.5 %)	130 (25.0 %)	0.835
Malignancy	23 (5.6 %)	33 (6.3 %)	0.638
Transplant	3 (0.7 %)	2 (0.4 %)	0.473
Chronic Kidney Disease	47 (11.4 %)	44 (8.4 %)	0.127
BMI kg/m^2^	29.17 (25.8–33.42)	29.29 (25.77–33.2)	0.8611
**Admission Characteristics**			
Oxygen saturation at presentation	94 (91–96)*	94 (91–96)†	0.1729
Respiratory Rate, respirations per minute	20 (19–24)	20 (18–24)	**0.0460**
Pulse, beats per minute	97.66±18.61	99.40±19.82	0.0858
Baseline Systolic BP, mmHg	134.83±20.84	132.41±21.87	**0.0435**
Baseline Diastolic BP, mmHg	76.66±12.62	76.59±14.22	0.4670
Temperature, degrees Celsius	37.65±0.82	37.72±0.94	0.1354
White blood cell count 10^3^/ul	6.9 (5.1–9.0) *N*=400	6.9 (5.1–9.3) *N*=500	0.5994
Absolute neutrophil count, 10^3^/ul	5.15 (3.6–7.05) *N*=388	5.4 (3.8–7.5) *N*=488	0.0838
Absolute lymphocyte count, 10^3^/ul	1 (0.7–1.3) *N*=388	0.9 (0.6–1.3) *N*=482	**0.0180**
Ferritin, ng ml^−1^	739 (379–1528) *N*=397	658 (336.2–1279) *N*=473	0.1304
d-Dimer, ng ml^−1^	341 (214–565) *N*=384	334 (215–587) *N*=435	0.7531
Troponin, ng ml^−1^	0.01 (0.01–0.02) *N*=389	0.015 (0.01–0.02) *N*=467	**0.0111**
Creatine Phosphokinase, U l^−1^	140 (68–330) *N*=343	151.5 (69.5–398.5) *N*=344	0.4371
Procalcitonin, ng l^−1^	0.12 (0.05–0.25) *N*=395	0.12 (0.06–0.43) *N*=478	**0.0493**
Creatinine, mg l^−1^	0.97 (0.8–1.34) *N*=400	0.99 (0.8–1.27) *N*=499	0.4140
C-Reactive Protein, mg l^−1^	104.95 (51.1–158.69) *N*=398	108.13 (53–157.11) *N*=480	0.9586
**Medications recorded during hospitalization**			
NSAID	53 (12.9 %)	74 (14.2 %)	0.563
Anticoagulant	402 (97.8 %)	511 (98.1 %)	0.772
ACE inhibitor or arb	138 (33.6 %	175 (33.7 %)	0.997
Beta Blocker	91 (22.1 %)	132 (25.3 %)	0.256
Calcium Channel Blocker	89 (21.7 %)	104 (20.0 %)	0.527
Corticosteroid	40 (9.7 %)	47 (9.0 %)	0.711

*Measured on supplemental oxygen for 86.4 %

†Measured on supplemental oxygen for 83.1 %.

In univariate analysis, the addition of zinc sulphate to hydroxychloroquine and azithromycin was not associated with a decrease in length of hospital stay, duration of mechanical ventilation, maximum oxygen flow rate, average oxygen flow rate, average fraction of inspired oxygen or maximum fraction of inspired oxygen during hospitalization (Table 2). In bivariate logistic regression analysis, the addition of zinc sulphate was associated with decreased mortality or transition to hospice (OR 0.511, 95 % CI 0.359–0.726), need for ICU (OR 0.545, 95 % CI 0.362–0.821) and need for invasive ventilation (OR 0.562, 95 % CI 0.354–0.891) (Table 3). However, after excluding all non-critically ill patients admitted to the intensive care unit, zinc sulphate no longer was found to be associated with a decrease in mortality (Table 3). Thus, this association was driven by patients who did not receive ICU care (OR 0.492, 95 % CI 0.303–0.799). We also found that the addition of zinc sulphate was associated with likelihood of discharge to home in univariate analysis (OR 1.56, 95 % CI 1.16–2.10) (Table 3). We performed a logistic regression model to account for the time-period when the addition of zinc sulphate to hydroxychloroquine plus azithromycin became utilized at NYULH. After adjusting for this date (25 March), we still found an association for likelihood of discharge to home (OR 1.53, 95 % CI 1.12–2.09) and decreased mortality or transition to hospice however the other associations were no longer significant (Table 3). The decrease in mortality or transition to hospice was most striking when considering only patients who were not admitted to the ICU (OR 0.449, *P* value: 0.002) (Table 3).

**Table 2.** Comparisons of continuous hospital outcomes. Data are represented median (IQR) and as mean±sd. Sample size is reported for each variable tested. *β* coefficients and *P* values were calculated using linear regression. *N* was specified for each comparison. *P* <0.05 was deemed significant

	Zinc	No zinc	*β* coefficient	*P* value
Length of ospital stay (in days)*	6 (4–9) *N*=411	6 (3–9) *N*=521	0.015	0.646
Duration of mechanical* ventilation (in days)	5 (3–8) *N*=33	5 (3–9) *N*=86	0.040	0.667
ICU duration (in days)*	4.85 (1.97–7.94) *N*=38	5.54 (2.65–9.32) *N*=82	−0.062	0.504
Oxygen flow rate maximum*	6 (3–15) *N*=353	6 (3–15) *N*=426	−0.015	0.679
Oxygen flow rate average*	3.05 (2.1–6.3) *N*=353	3.5 (2.5–7.5) *N*=426	−0.062	0.082
Fraction of inspired oxygen, average	61.52±32.03 *N*=107	65.26±34.48 *N*=117	−0.056	0.402
Fraction of inspired oxygen, maximum	74.94±35.75 *N*=107	71.98±35.85 *N*=117	0.041	0.538

*Variables were log transformed for regression analysis. Intensive care unit is abbreviated to ICU.

**Table 3.** Comparison of categorical hospital outcomes. Data are represented as *N* (%). *P* values were calculated using logistic regression or multivariate logistic regression adjusting for patient admission after 25 March as a categorical variable. *P* <0.05 was deemed significant. *N* was specified for subgroup analyses. Intensive care unit is abbreviated to ICU

	Discharged home	Needed ICU	Needed invasive Ventilation	Expired/hospice	Expired/hospice*	Expired/hospice†
**Zinc** *N*=411	317 (77.1 %)	38 (9.2 %)	29 (7.1 %)	54 (13.1 %)	28 (73.6 %) *N*=38	26 (6.9 %) *N*=373
**No Zinc** *N*=521	356 (68.3 %)	82 (15.7 %)	62 (11.9 %)	119 (22.8 %)	61 (74.4 %) *N*=82	58 (13.2 %) *N*=439
**Odds Ratio**	1.56	0.545	0.562	0.511	0.964	0.492
**95 % Confidence Interval**	1.16–2.10	0.362–0.821	0.354–0.891	0.359–0.726	0.401–2.31	0.303–0.799
***P* value**	**0.003**	**0.004**	**0.014**	**<0.0001**	0.934	**0.004**
**Adjusted Odds Ratio**	1.53	0.733	0.804	0.559	1.03	0.449
**Adjusted 95 % Confidence Interval**	1.12–2.09	0.471–1.14	0.487–1.33	0.385–0.811	0.404–2.64	0.271–0.744
**Adjusted *P* value**	**0.008**	0.168	0.396	**0.002**	0.947	**0.002**

*After excluding all non-ICU patients.

†After excluding all ICU patients.

## Discussion

While practicing at the epicentre of the pandemic in the United States, we were faced with unprecedented challenges of adopting investigational therapies quickly into clinical practice. Initially, antiviral options at our institution consisted of clinician preference for either ritonavir/lopinavir or hydroxychloroquine plus azithromycin. After the findings of ritonavir/lopinavir, we noticed an increase in the use of hydroxychloroquine plus azithromycin [16]. Our providers within the infectious diseases division, clinical pharmacy and hospitalists discussed the use of zinc sulphate as an addition to hydroxychloroquine, based on the potential synergistic mechanism, and low risk of harm associated with this therapy.

There has been significant interest in the use of zinc sulphate to treat and prevent COVID-19 infection and its use is being considered in several trials (NCT04326725, NCT04377646, NCT04370782, NCT04373733, NCT04351490) [9, 17, 18]. To our knowledge, we provide the first *in vivo* evidence on the efficacy of zinc sulphate in addition to hydroxychloroquine in COVID-19 patients. The main finding of this study is that the addition of zinc sulphate to hydroxychloroquine and azithromycin was found to be associated with a decrease in mortality or transition to hospice among patients who did not require ICU level of care, but this association was not seen for patients who were treated in the ICU. This result may reflect one of the proposed mechanisms by which zinc sulphate may provide protection against COVID-19. Zinc has been shown to reduce SARS-CoV RNA-dependent RNA polymerase activity *in vitro* [14]. As such, zinc may have a role in preventing the virus from progressing to severe disease, but once the aberrant production of systemic immune mediators is initiated, known as the cytokine storm, the addition of zinc may no longer be effective [19]. To further examine the ability of zinc to prevent viral replication and disease progression, future studies should measure viral RNA levels in clinical specimens before and after zinc administration. Our findings suggest a potential protective effect of zinc, potentially enhanced by a therapeutic synergistic mechanism of zinc sulphate with hydroxychloroquine, if used early on in presentation with COVID-19.

After adjusting for the timing of zinc sulphate treatment, the negative associations between zinc and the need for ICU and invasive ventilation were no longer significant but we did still observe a trend. This observation may be because patients with COVID-19 were initially sent to the ICU quicker, but as time went on and resources became more limited, clinicians began treating COVID-19 patients on general medicine floors for longer periods of time before escalating to the ICU. Future studies are needed to confirm or refute the hypothesis that the addition of zinc sulphate to a zinc ionophore such as hydroxychloroquine may reduce the need for ICU care in patients with COVID-19.

This study has several limitations. First, this was an observational retrospective analysis that could be impacted by confounding variables. This is well demonstrated by the analyses adjusting for the difference in timing between the patients who did not receive zinc and those who did. In addition, we do not know whether the observed added benefit of zinc sulphate to hydroxychloroquine and azithromycin on mortality would have been seen in patients who took zinc sulphate alone or in combination with just one of those medications since few patients at our hospitals received zinc sulphate as stand-alone therapy. The optimal dose and formulation of zinc supplementation necessary to inhibit RNA-dependent RNA polymerase activity of coronaviruses also remains unknown. Early reports suggested higher doses may be necessary, ranging from 50 to 150 mg elemental zinc/day, and such doses have been proved to be safe for short periods of time when used for other viruses or in improving immune reconstitution [20–23]. In addition, although other formulations of zinc supplementation exist, in the form of acetate or gluconate, our hospital formulary had sulphate, which generally has a higher elemental amount of zinc per tablet. Therefore, further investigation is required to determine whether sulphate is the preferred formulation when used to treat coronaviruses, or if another formulation may be better tolerated. Given the recent concern regarding the potential for side effects associated with hydroxychloroquine, future studies should examine whether zinc sulphate would provide benefit as a stand-alone therapy or in combination with another zinc ionophore. In addition, as zinc is hypothesized to inhibit viral RNA polymerase, future studies are needed to determine if zinc synergistically acts with other antiviral medications. We also do not have data on the time at which the patients included in the study initiated therapy with hydroxychloroquine, azithromycin and zinc. Those drugs would have been started at the same time as a combination therapy, but the point in clinical disease at which patients received those medications could have differed between our two groups. Finally, the cohorts were identified based on medications ordered rather than confirmed administration, which may bias findings towards favouring equipoise between the two groups.

## Conclusion

Zinc sulphate added to hydroxychloroquine and azithromycin associates with a decrease in mortality or transfer to hospice among patients who do not require ICU level of care and an increased likelihood to be discharged directly home from the hospital. In light of study limitations, this study alone is not sufficient to guide clinical practice. Rather, these findings suggest a potential role for zinc sulphate in COVID-19 patients and support the initiation of future randomized clinical trials investigating zinc sulphate against COVID-19.
